# Obstruction of the Bowels

**Published:** 1853-03

**Authors:** 


*Obstruction of the Bowels.* Two Cases reported by E. D. Fenner, M. D.

Case 1. A few weeks since, I was requested by Dr. Moss, of this city, to assist him in the post-mortem examination of a mulatto man aged about 35 years, who, after suffering repeated attacks of obstruction of the bowels, accompanied by great pain and stercoraceous vomiting, finally sank and died. He had suffered three attacks within the month previous to death. Dr. M. had attended him in several of them, and only succeeded in relieving him with great difficulty by means of free cupping, the warm bath, and Croton oil. These means succeeded in opening his bowels in his last attack, but he did not recuperate afterward. On examination after death, we found a *small piece of bone* lodged in the lower portion of the ilium. It had excited inflammation and *thickening of the intestinal walls* to such extent, as to cause an almost impermeable stricture of the canal. Here was the cause of death. The piece of bone was only about *three-fourths of an inch in length*, and rather flat. The ends were not sharp, and the only wonder is, that it had not passed without difficulty.

Now let us see how much larger an amount of foreign substance did pass the entire extent of the alimentary canal, till it reached the anus, where it was impeded by the sphincter, and had to be removed mechanically.

Case 2. On the 11th September, 1852, I was called to see a white female child, aged about two and a half years. I was told that she had diarrhœa with prolapsus of the rectum. No assignable cause was mentioned at the time. About three months previously, I had attended this child for an obstinate attack of diarrhœa, and relieved her entirely.

On this morning, the child did not appear to be much sick, I advised a little Hydrarg. C. Creta, to be followed by a dose of castor oil. A few hours afterward I was sent for, and informed that a piece of cork had been discovered in the child's anus. Upon reaching the patient, I found this to be the case; a large piece of cork was plainly visible. I readily succeeded in removing it with my finger; but this was not all. I continued to take away piece after piece, until I removed *nearly a handful.* The operation gave considerable pain, and caused slight hæmorrhage, but I removed all I could reach. I then prescribed a dose of castor oil, which produced a copious operation, and gave complete *relief.* A considerable quantity of cork came away some days afterward. We were then informed by a larger sister of this child, that she had often observed her with cork in her mouth, but did not know that she had swallowed it. Thus it is evident that this large amount of *cork*, some of the pieces as big as the end of my thumb, had been swallowed, and traversed the alimentary canal as low as the anus. There were, perhaps, a dozen pieces, twice as large as the piece of bone that caused the death of the man first mentioned. The quantity of cork passed completely filled a common match box.—*New Orleans Monthly Med. Register.*

